# Beyond Implant Design: Surgical Principles and Early Outcomes of Patient-Specific Scapular Reconstruction After Total Scapulectomy

**DOI:** 10.3390/curroncol33090565

**Published:** 2026-09-17

**Authors:** Adyb-Adrian Khal, Ruxandra Florina Bodog, Florian Dorel Bodog, Ovidiu-Gogu Cacuci

**Affiliations:** 1 Faculty of Medicine and Pharmacy, University of Oradea, 410087 Oradea, Romania; 2 Department of Orthopaedics, Pelican Medicover Hospital, 410450 Oradea, Romania; 3 Department of Plastic Surgery, Pelican Medicover Hospital, 410450 Oradea, Romania

**Keywords:** scapulectomy, scapular reconstruction, patient-specific implant, 3D printing, bone sarcoma, limb salvage

## Abstract

**Background/Objectives**: Total scapulectomy for malignant scapular tumors remains a major reconstructive challenge because resection removes the osseous framework, muscular insertions, and biomechanical support required for shoulder function. Patient-specific three-dimensional (3D)-printed implants may improve anatomical reconstruction, but the indications and surgical principles underlying successful total scapular replacement remain poorly defined. **Methods**: This retrospective single-center case series included the first three consecutive patients who underwent total scapulectomy followed by immediate reconstruction using a patient-specific 3D-printed scapular prosthesis between January 2024 and December 2025. Clinical, radiographic, oncological, and functional outcomes were reviewed. Functional outcome was assessed using the Musculoskeletal Tumor Society (MSTS) score and active shoulder range of motion. **Results**: The cohort included two women and one man with a median age of 53 years (range, 43–58 years). Diagnoses were chondrosarcoma in two patients and osteosarcoma in one. All tumors were Enneking stage IIB. Negative margins were achieved in all patients, and the axillary nerve and principal shoulder-girdle stabilizers were preserved in every case. Median follow-up was 13 months (range, 12–27 months). At latest follow-up, two patients had no evidence of disease and one was alive with disease after developing pulmonary metastases. The median MSTS score was 25/30 (range, 21–26). Median active elbow flexion, abduction, and forward flexion measured 88°, 80°, and 80°, respectively. Two patients experienced complications: one clavicular fracture and one aseptic wound dehiscence; neither resulted in implant failure or required revision of the reconstruction. **Conclusions**: Patient-specific total scapular reconstruction may provide satisfactory early radiographic and functional outcomes in carefully selected patients, although outcomes remain variable depending on individual patient and tumor characteristics. Implant design, patient selection, preservation of the axillary nerve and viable innervated shoulder-girdle musculature, and soft-tissue reconstruction should be considered complementary components of this reconstructive strategy.

## Simple Summary

Reconstruction after complete removal of the scapula for a malignant tumor is one of the most demanding procedures in orthopedic oncology. Although patient-specific implants have expanded reconstructive options, it remains unclear which patients are most likely to benefit from these complex procedures. We report the early outcomes of three patients treated with patient-specific scapular reconstruction after total scapulectomy and describe the surgical principles that guided our treatment strategy. Our experience suggests that implant design, careful patient selection, preservation of key nerves and viable musculature, and meticulous soft-tissue reconstruction should be considered complementary components of successful reconstruction. These findings highlight that patient-specific implants should be considered part of a broader biological and surgical strategy rather than a solution on their own. This study may help surgeons refine the indications for patient-specific scapular reconstruction and improve functional outcomes after this rare and challenging procedure.

## 1. Introduction

Primary malignant tumors of the scapula are rare but represent one of the most challenging conditions in musculoskeletal oncology because of the complex anatomy of the shoulder girdle and the intimate relationship between the scapula, surrounding musculature, and major neurovascular structures [1,2,3,4,5,6]. Since the development of limb-salvage surgery and shoulder girdle resection classifications, total scapulectomy has progressively become an accepted alternative to forequarter amputation in appropriately selected patients, providing oncological control while preserving elbow, wrist, and hand function [1,2,3,4,5,6,7,8]. However, complete removal of the scapula simultaneously eliminates the osseous support, muscular insertions, and biomechanical stability required for normal shoulder function, making reconstruction particularly challenging [2,4,5,6,7,8].

Numerous reconstructive techniques have been described following total scapulectomy, including no reconstruction, humeral suspension, mesh reconstruction, osteoarticular allografts, extracorporeally irradiated autografts, allograft-prosthetic composites, conventional scapular prostheses, and constrained reverse shoulder arthroplasty [3,4,5,7,8,9,10,11,12,13,14]. Although each technique has specific advantages and limitations, no reconstructive strategy has demonstrated clear superiority, and postoperative shoulder function remains highly variable. Consequently, reconstruction continues to be individualized according to tumor extent, remaining soft tissues, abductor mechanism preservation, surgeon experience, and implant availability rather than well-defined indications [1,4,5,6,7,8,9,10,11,12,13,14].

Recent advances in computer-assisted planning, three-dimensional imaging, and additive manufacturing have enabled the development of patient-specific scapular and glenoid prostheses capable of reproducing complex anatomy while incorporating individualized fixation methods and dedicated sites for soft-tissue reattachment [12,13,14,15,16]. Early clinical experience has demonstrated encouraging functional and radiological outcomes, suggesting that patient-specific reconstruction may improve shoulder stability and upper-limb function after total or partial scapulectomy [12,13,14,15,16]. The available evidence remains limited to isolated case series and small retrospective series, with considerable heterogeneity in implant design, surgical technique, and outcome assessment [12,13,14,15,16]. Although previous studies have described custom prosthetic designs, surgical techniques, and functional outcomes [12,13,14,15,16], the interplay between patient selection, preservation of viable innervated shoulder-girdle musculature, implant design, and soft-tissue reconstruction has been less explicitly addressed as an integrated reconstructive strategy. The present technical case series therefore focuses on how these complementary factors were incorporated into surgical decision-making and reconstruction in three consecutive patients undergoing patient-specific total scapular replacement.

## 2. Materials and Methods

### 2.1. Study Design

This retrospective single-center case series included the first three consecutive patients who underwent limb-salvage surgery with reconstruction using a patient-specific three-dimensional (3D)-printed scapular implant following oncologic resection between January 2024 and December 2025.

### 2.2. Patient Selection

Patients were eligible if they underwent total scapulectomy for a primary malignant, locally aggressive, or metastatic scapular tumor followed by immediate reconstruction using a patient-specific 3D-printed implant. Patients reconstructed using conventional techniques, including humeral suspension, allograft reconstruction, or modular prosthetic replacement, were not included. Conversely, patient-specific total scapular reconstruction was not considered appropriate when achieving adequate oncological margins required sacrifice of the axillary nerve or extensive resection of the principal innervated shoulder-girdle musculature necessary for functional soft-tissue reconstruction.

Demographic characteristics, tumor diagnosis, previous oncological treatments, preoperative imaging, operative reports, pathological findings, postoperative complications, oncological outcomes, radiographic follow-up, and functional outcomes were retrospectively collected from the institutional medical records.

### 2.3. Preoperative Evaluation and Virtual Surgical Planning

All patients underwent standardized preoperative evaluation including plain radiographs, computed tomography (CT), and magnetic resonance imaging (MRI). Thin-slice CT scans were used to accurately define the osseous anatomy and generate three-dimensional reconstructions, whereas MRI was used to assess intraosseous tumor extension, soft-tissue involvement, and the relationship of the tumor to adjacent neurovascular structures. Additional staging investigations were performed according to the underlying tumor histology.

Each case was discussed within a dedicated multidisciplinary musculoskeletal oncology team to determine the optimal treatment strategy. Limb-salvage surgery was proposed whenever complete tumor resection with adequate oncological margins was considered achievable while preserving a functional upper extremity.

Virtual surgical planning was performed collaboratively by the orthopaedic oncology surgeons and biomedical engineers. CT and MRI datasets were integrated to define patient-specific osteotomy planes and optimize resection margins while preserving uninvolved anatomical structures whenever oncologically appropriate. The interval between completion of imaging and definitive surgery was approximately 6–8.5 weeks, reflecting virtual planning, implant manufacturing, sterilization, and surgical scheduling. During this period, patients with osteosarcoma continued neoadjuvant chemotherapy, whereas those with chondrosarcoma underwent repeat imaging to exclude clinically significant tumor progression before definitive surgery.

### 2.4. Implant Design and Manufacturing

Patient-specific scapular implants were designed through close collaboration between the treating orthopaedic oncology surgeons and biomedical engineers. The implant geometry was individually adapted to each patient’s anatomy and planned resection with the objectives of restoring the contour of the shoulder girdle, providing stable skeletal reconstruction, and maximizing the available sites for soft-tissue reattachment.

Because total scapulectomy inevitably requires sacrifice of several dynamic stabilizers of the shoulder, implant design prioritized reconstruction of shoulder contour, stable skeletal reconstruction, and optimization of soft-tissue reattachment rather than anatomical recreation of glenohumeral biomechanics. The final implant design was established following iterative discussions between the surgical and engineering teams. Three-dimensional digital models were reviewed to validate implant fit, fixation strategy, prosthetic orientation, and the location of dedicated soft-tissue attachment sites before manufacturing. Three patient-specific scapular implants were additively manufactured (3D-printed) from titanium alloy by companies specializing in custom oncological reconstruction (Waldemar Link GmbH & Co. KG, Hamburg, Germany, *n* = 2; Implantcast GmbH, Buxtehude, Germany, *n* = 1). All implants were developed according to the same surgical principles but differed in design: two incorporated porous surfaces at selected bone-contact interfaces to promote biological integration, whereas one used a non-porous interface.

Dedicated suture holes were incorporated into each implant to facilitate anatomical reinsertion of all preserved muscles and tendons whenever oncologically feasible. Soft-tissue preservation and reconstruction were considered integral components of the reconstructive strategy, aiming to optimize postoperative shoulder stability and function.

Because all patients underwent total scapulectomy, patient-specific scapular cutting guides were not required. However, patient-specific humeral cutting guides were used to reproduce the planned humeral osteotomy and facilitate accurate implantation of the humeral prosthetic component according to the preoperative virtual surgical plan.

### 2.5. Surgical Technique

All procedures were performed with the patient in the lateral decubitus position using a combined anterior and posterior approach to the shoulder girdle. The surgical strategy was planned to allow safe vascular control, en bloc tumor resection, and immediate reconstruction with the patient-specific scapular implant.

The first step consisted of careful identification and dissection of the major vascular structures and preservation of the neurovascular bundle. Particular attention was paid to preserving the axillary nerve, as functional reconstruction with a total scapular prosthesis is not feasible without an intact neurovascular supply. The scapula was progressively mobilized by detaching the muscular insertions from inferior to superior while preserving all uninvolved muscles whenever oncologically possible.

The scapula was removed en bloc according to the preoperative oncological plan. Humeral preparation was performed using patient-specific cutting guides to reproduce the planned humeral osteotomy and facilitate accurate implantation of the humeral prosthetic component.

**Figure 1:**
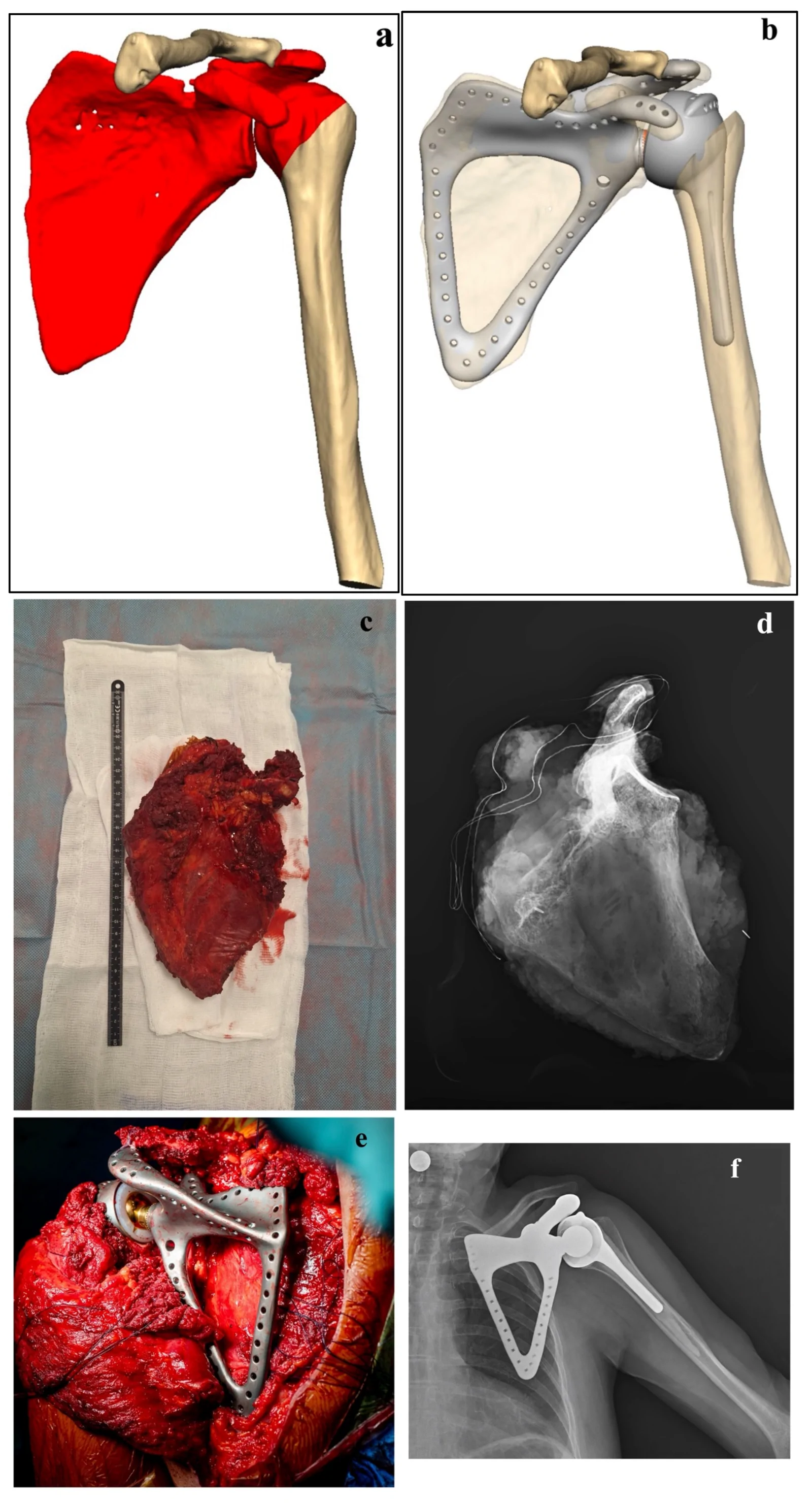
**Preoperative planning, oncological resection, and immediate reconstruction following patient-specific total scapular replacement.** A 53-year-old woman diagnosed with Enneking stage IIB osteosarcoma of the left scapula. (**a**) Preoperative three-dimensional reconstruction demonstrated the tumor extent and served as the basis for virtual surgical planning. (**b**) A patient-specific three-dimensional (3D)-printed titanium scapular prosthesis was designed according to the planned oncological resection. Following en bloc total scapulectomy, the resected specimen was evaluated macroscopically (**c**) and radiographically (**d**) to confirm complete tumor excision. Immediate reconstruction was then performed using the customized scapular prosthesis combined with reverse shoulder arthroplasty, clavicular fixation, and anatomical soft-tissue reconstruction (**e**). (**f**) Six-month postoperative anteroposterior radiograph demonstrating the reconstructed shoulder girdle.

All reconstructions incorporated a constrained reverse shoulder arthroplasty. Humeral stem fixation was cemented in two patients and press-fit in one, with the fixation strategy selected according to patient age. The patient-specific scapular implant was then positioned and stabilized. Clavicular fixation was selected according to the planned reconstructive strategy, using screw fixation for the two LINK (Waldemar Link GmbH & Co. KG, Hamburg, Germany,) implants and FiberWire (Arthrex GmbH, Munchen, Germany) suspension for the Implantcast implant. The principal steps of patient-specific total scapular reconstruction are illustrated in Figure 1.

Soft-tissue reconstruction represented a critical step of the procedure. All preserved muscles were reattached to the prosthesis through dedicated fixation holes using nonabsorbable sutures. Particular attention was given to preserving and reconstructing the trapezius, deltoid, rhomboids, serratus anterior, and latissimus dorsi, as these muscles provide soft-tissue coverage, suspend the prosthesis, and contribute to functional shoulder stability.

### 2.6. Postoperative Rehabilitation

Postoperatively, the operated upper limb was immobilized for four weeks using an abduction brace maintaining the shoulder at approximately 45° of abduction to protect the soft-tissue reconstruction and minimize stress on the prosthesis. During the immobilization period, active and passive range-of-motion exercises of the wrist and hand were initiated immediately after surgery. Active and passive elbow mobilization was also permitted from the immediate postoperative period while maintaining shoulder immobilization.

After four weeks, the abduction brace was discontinued and patients underwent a standardized rehabilitation program supervised by experienced physiotherapists. Rehabilitation focused on the progressive restoration of passive and active shoulder range of motion, followed by gradual muscle strengthening and functional recovery according to individual clinical progress and soft-tissue healing.

### 2.7. Outcome Measures

Patients underwent regular clinical, radiographic, and oncological follow-up according to institutional practice. Functional outcome was assessed using the Musculoskeletal Tumor Society (MSTS) score together with active shoulder range of motion measured clinically using a goniometer, including active forward flexion, abduction, internal rotation, and external rotation. Internal and external rotation deficits were defined as the loss of motion relative to predefined normal range-of-motion values. Postoperative function was assessed clinically; no electromyographic or formal postoperative neurophysiological assessment was performed.

Radiographs were independently reviewed by the treating surgeons for implant position, prosthetic stability, radiolucent lines suggestive of aseptic loosening, implant migration, clavicular fixation integrity, structural implant failure, prosthetic dislocation, and periprosthetic fracture. Because no validated radiographic classification currently exists for patient-specific total scapular prostheses, implant stability was assessed descriptively based on the absence of these radiographic signs during serial follow-up examinations. Additional CT imaging was obtained whenever clinically indicated.

Oncological follow-up included assessment of surgical margins, local recurrence, distant metastasis, and disease status at the latest follow-up. All postoperative complications and reoperations were prospectively recorded and retrospectively reviewed.

### 2.8. Statistical Analysis

Given the limited sample size, only descriptive statistics were performed. Continuous variables are presented as median (range) or mean (range), as appropriate, whereas categorical variables are presented as frequencies and percentages. Statistical analyses were performed using IBM SPSS Statistics version 29.0 (IBM Corp., Armonk, NY, USA).

## 3. Results

### 3.1. Patient Characteristics

Between January 2024 and December 2025, three consecutive patients underwent total scapulectomy with immediate reconstruction using a patient-specific three-dimensional (3D)-printed scapular prosthesis (Table 1). The cohort comprised two women and one man with a median age of 53 years (range, 43–58 years). Histological diagnoses included two grade 2 chondrosarcomas and one conventional osteosarcoma.

All tumors were classified as Enneking stage IIB, and histological diagnosis was established by preoperative biopsy in every case. None of the patients presented with distant metastases at diagnosis. No patient had undergone previous tumor resection or radiotherapy. The patient with conventional high-grade osteosarcoma received neoadjuvant chemotherapy followed by wide tumor resection and postoperative adjuvant chemotherapy.

The median clinical follow-up was 13 months (range, 12–27 months). At the latest follow-up, two patients had no evidence of disease (NED), whereas one patient was alive with disease (AWD) after developing pulmonary metastases.

### 3.2. Surgical Characteristics

All patients underwent limb-salvage surgery consisting of total scapulectomy and immediate reconstruction with a patient-specific 3D-printed scapular prosthesis combined with reverse shoulder arthroplasty. In all three patients, the axillary nerve was preserved, together with viable, innervated portions of the deltoid, trapezius, rhomboids, serratus anterior, and latissimus dorsi sufficient for functional soft-tissue reconstruction and reattachment to the prosthesis. The rotator cuff and suprascapular neurovascular bundle were sacrificed as part of the oncological resection. All preserved muscles were subsequently reattached to the dedicated fixation sites of the prosthesis. Complete tumor excision with negative (R0) margins was achieved in every case.

The median operative time was 250 min (range, 240–310 min), and the median estimated intraoperative blood loss was 800 mL (range, 800–1000 mL). Two custom implants were secured to the clavicle using screw fixation, whereas one implant was suspended from the clavicle using FiberWire fixation. Two humeral stems were cemented and one was implanted using a press-fit technique. Anatomical soft-tissue reconstruction was successfully completed in all patients.

### 3.3. Oncological Outcomes

Negative surgical margins were obtained in all patients. During follow-up, one patient developed pulmonary metastases. The remaining two patients remained free of local recurrence and distant metastases throughout follow-up.

### 3.4. Functional and Radiographic Outcomes

At the latest follow-up, the median Musculoskeletal Tumor Society (MSTS) score was 25/30 (range, 21–26). Median active elbow flexion was 88° (range, 84–100°), median abduction was 80° (range, 45–83°), and median forward flexion was 80° (range, 55–81°). Internal rotation was the most restricted movement, with deficits ranging from 30° to 70°, whereas external rotation deficits ranged from 10° to 45°.

**Figure 2:**
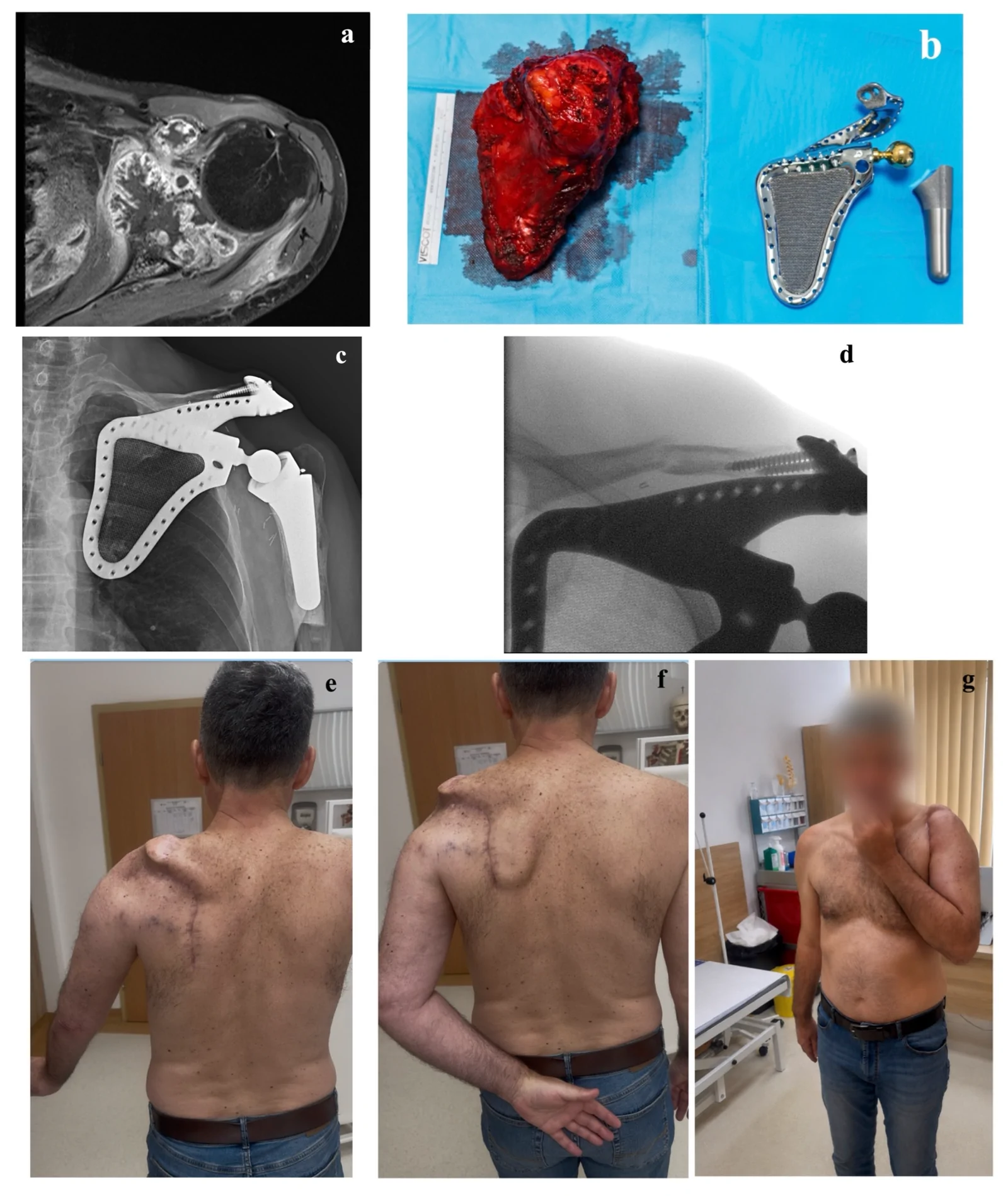
**Clinical course of a patient following patient-specific total scapular reconstruction.** A 58-year-old man diagnosed with grade II chondrosarcoma of the left scapula. (**a**) Preoperative axial T2-weighted magnetic resonance imaging demonstrated the extent of the tumor. (**b**) Following oncological total scapulectomy, the resected specimen is shown alongside the patient-specific three-dimensional (3D)-printed titanium scapular prosthesis and reverse shoulder arthroplasty components before implantation. (**c**) Immediate postoperative anteroposterior radiograph demonstrated satisfactory reconstruction with restoration of the shoulder girdle anatomy. (**d**) At 7 months postoperatively, a clavicular fracture occurred without displacement of the prosthesis or loss of reconstruction. Although the patient subsequently developed pulmonary metastases, the reconstruction remained without implant failure or the need for revision surgery. At the latest follow-up, active shoulder abduction (**e**), functional internal rotation (**f**), and preserved hand-to-mouth function (**g**) were maintained despite systemic disease progression.

All three patients retained useful hand, wrist, and elbow function, allowing independent performance of activities of daily living (Figure 2).

Persistent shoulder pain was observed only in the patient who subsequently developed pulmonary metastases. The remaining two patients were pain-free and achieved satisfactory functional use of the operated upper limb during activities of daily living.

During the available short-term follow-up, serial radiographs showed no evidence of implant migration, progressive radiolucent lines suggestive of aseptic loosening, clavicular fixation failure, structural implant failure, prosthetic dislocation, or periprosthetic fracture.

### 3.5. Complications

No intraoperative complications occurred. Two patients developed postoperative complications: one aseptic wound dehiscence at 4 weeks, which healed without implant removal or deep infection, and one spontaneous midshaft clavicular fracture at 7 months. The fracture was treated conservatively and remained ununited at the latest follow-up, but had no clinical impact and did not compromise implant stability or require revision. No prosthetic infection, dislocation, loosening, or structural implant failure occurred during follow-up.

## 4. Discussion

Reconstruction of the shoulder girdle after total scapulectomy remains challenging because resection removes not only the scapular bone, but also the anatomical platform for muscular insertion, upper-limb suspension, and shoulder biomechanics [2,3,4,5,7,8]. Custom-made scapular prostheses, constrained reverse shoulder arthroplasty, and patient-specific three-dimensional (3D)-printed implants have expanded reconstructive possibilities [9,12,13,14,15,17,18,19]. However, the evidence remains limited to small retrospective series and case reports, and the optimal indications for anatomical total scapular reconstruction remain poorly defined [8,14,19,20].

The present study describes our initial experience with patient-specific total scapular reconstruction after oncologic resection. All patients were treated according to the same reconstructive philosophy: complete tumor resection with negative margins, preservation of the axillary nerve and principal dynamic stabilizers, and immediate patient-specific reconstruction incorporating clavicular suspension, reverse shoulder articulation, and soft-tissue reattachment. At a median follow-up of 13 months, no radiographic evidence of aseptic loosening, prosthetic dislocation, clavicular fixation failure, or structural implant failure was observed during the available short-term follow-up. The median MSTS score was 25/30, suggesting that anatomical reconstruction may provide encouraging early function when strict oncological and biological prerequisites are fulfilled.

Reconstruction after total scapulectomy has evolved from limb preservation toward anatomical and functional restoration. Humeral suspension and non-reconstructive techniques remain valuable because they are relatively simple and preserve distal upper-limb function, although active shoulder motion is generally limited [2,3,4,5,7,8]. Custom scapular prostheses and reverse shoulder reconstruction may improve shoulder stability, contour, and active upper-limb positioning in selected patients [8,9,12,13,14,15,17,18,19,20]. These procedures should be regarded as complementary solutions whose indications depend on tumor extent and remaining functional anatomy.

The present case series suggests that implant design, patient selection, preservation of the biological environment, and soft-tissue reconstruction should be considered complementary components of patient-specific total scapular reconstruction. Preservation of the axillary nerve together with viable innervated portions of the deltoid, trapezius, rhomboids, serratus anterior, and latissimus dorsi was an integral component of our patient-selection and reconstructive strategy, as these structures contribute to the soft-tissue envelope, dynamic stability, and muscular balance required to activate and stabilize the prosthesis [2,4,6,12,13,21,22]. However, because all patients were selected according to these criteria, the present series cannot establish the independent contribution of preserving these structures to functional outcome. Compared with non-anatomical reconstruction or humeral suspension, the intended advantage of a patient-specific implant is to reproduce scapular morphology and glenoid orientation while integrating clavicular suspension, constrained reverse shoulder reconstruction, and dedicated soft-tissue attachment sites within a single construct [9,12,13,14,15,16,17,18,19,20,23]. When these structures cannot be preserved because of tumor extension, humeral suspension or another non-anatomical reconstruction may provide a more predictable alternative.

Soft-tissue reconstruction therefore represents a central technical component of this procedure. In all patients, viable innervated portions of the retained shoulder-girdle musculature were reattached to dedicated prosthetic fixation holes using nonabsorbable sutures to restore soft-tissue continuity and provide functional coverage [2,6,12,13,21,22].

Reverse shoulder arthroplasty may improve stability and shift shoulder function toward the deltoid when the rotator cuff or glenoid is sacrificed [6,21,22,24,25]. After total scapulectomy, however, reverse shoulder arthroplasty alone is insufficient. It requires a stable reconstructed scapular body, adequate clavicular suspension, preserved deltoid function, and appropriate soft-tissue tensioning [6,13,15,17,21,22,24,25]. Patient-specific implants may facilitate this by allowing the surgical and engineering teams to plan the relationship between the clavicle, humerus, reverse shoulder articulation, and muscle attachment points before surgery. The two implant systems used different technical configurations, including porous versus non-porous bone-contact interfaces and screw fixation versus FiberWire clavicular suspension. Given the cohort size and short follow-up, these differences are reported descriptively only, and no comparison of fixation performance, durability, or failure mechanisms between implant designs can be made.

The early functional outcomes in this series are consistent with outcomes reported in the available literature. Functional recovery was not uniform, as Patient 1 achieved a lower MSTS score despite a stable reconstruction. Tumor biology, postoperative complications, systemic disease progression, and individual patient factors are all likely to influence the final functional outcome. Historical total scapulectomy series generally report modest shoulder function, even when elbow, wrist, and hand function are preserved [2,3,4,5,7]. Contemporary comparative studies suggest that prosthetic or bony reconstruction may improve active motion and MSTS scores compared with humeral suspension or non-reconstruction in selected patients, although outcomes remain heterogeneous [4,7,8,14,20]. In the present study, the median MSTS score was 25/30, with median active elbow flexion of 88°, abduction of 80°, and active forward flexion of 80°. These results should be interpreted cautiously because of the small cohort, short follow-up, and absence of objective postoperative assessment of individual muscle or nerve function; consequently, no specific anatomical mechanism can be inferred from the observed functional outcomes.

Complications remain a major concern after scapular reconstruction, with wound complications, infection, instability, clavicular problems, local recurrence, systemic progression, and mechanical failure reported in the literature [4,7,8,13,14,20]. In our series, two of three patients experienced complications: one aseptic wound dehiscence that healed without implant removal and one spontaneous midshaft clavicular fracture treated conservatively, which remained ununited but did not compromise implant stability or require revision. Although neither complication resulted in reconstructive failure, their occurrence highlights the morbidity associated with these complex procedures. One patient also developed pulmonary metastases, representing oncological progression rather than reconstructive failure.

This study has several limitations. First, it is a retrospective single-center case series of only three patients. Second, the median follow-up of 13 months precludes conclusions regarding long-term oncological outcomes, implant survival, biological integration, late infection, and mechanical durability. Third, there was no control group treated with humeral suspension, mesh reconstruction, or non-reconstructive techniques. In addition, preservation of the axillary nerve and sufficient viable innervated shoulder-girdle musculature formed part of the selection criteria for this reconstruction; therefore, the present series cannot determine the independent effect of preserving these structures on functional outcome. Fourth, two implant manufacturers were used, although all reconstructions followed the same surgical principles. Finally, functional assessment was limited to postoperative MSTS score and range of motion, as standardized preoperative functional measurements were not consistently available. Despite these limitations, the series is homogeneous in indication and surgical philosophy and provides a detailed technical description of patient-specific total scapular reconstruction after oncological resection.

## 5. Conclusions

Patient-specific total scapular reconstruction appears to be a promising limb-salvage option after oncological total scapulectomy in carefully selected patients. In this initial series, the procedure provided encouraging early functional and radiographic outcomes when incorporated into a reconstructive strategy based on appropriate patient selection, preservation of viable innervated shoulder-girdle structures, and meticulous soft-tissue reconstruction. The patient-specific implant may facilitate restoration of shoulder contour, clavicular suspension, reverse shoulder reconstruction, and anatomical soft-tissue reattachment; however, the present findings should be interpreted in the context of the small cohort and short follow-up. Larger multicenter studies with longer follow-up are required to better define patient-selection criteria, long-term implant durability, and functional outcomes.

## Figures and Tables

**Table 1:** Demographic characteristics, surgical details, functional outcomes, complications, and oncological outcomes of the three patients undergoing patient-specific total scapular reconstruction.

Patient	Age/Sex/Lesion	Stem and Clavicular Fixation	Complications (x, Time)	FU (Months)	MSTS	Active ROM	Pain	Oncological Status
1	58, M, ChS	Cemented, Screws	Fx, 7 months Met, 10 months	27	21	ABD 45, EF 84, AFF 55, IRD 70, ERD 45	Yes	AWD
2	43, F, ChS	Pressfit, Screws	Aswd, 4 weeks	13	25	ABD 80, EF 88, AFF 81, IRD 40, ERD 20	No	NED
3	53, F, OS	Cemented, Fiberwire	-	12	26	ABD 83, EF 100, AFF 80, IRD 30, ERD 10	No	NED

FU = Follow-up, MSTS = Musculoskeletal Tumor Society Score, ROM = Range of motion, M = Male, F = Female, ChS = Chondrosarcoma, OS = Osteosarcoma, Fx = Fracture, Met = Metastasis, Aswd = Aseptic wound dehiscence, ABD = Abduction, EF = Elbow Flexion, AFF = Active forward flexion, IRD = Internal Rotation Deficit, ERD = External Rotation Deficit, AWD = alive with disease, NED = no evidence of disease.

## Data Availability

On request from the corresponding author, the data are not publicly available due to privacy and ethical reasons.

## Abbreviations

The following abbreviations are used in this manuscript:

FU	Follow-up
MSTS	Musculoskeletal Tumor Society Score
ROM	Range of motion
M	Male
F	Female
ChS	Chondrosarcoma
OS	Osteosarcoma
Fx	Fracture
Met	Metastases
Aswd	Aseptic Wound Dehiscence
ABD	Abduction
EF	Elbow Flexion
AFF	Active Forward Flexion
IRD	Internal Rotation Deficit
ERD	External Rotation Deficit
AWD	Alive with Disease
NED	No evidence of disease
